# Facilitating factors for seeking help for mental health problems among Norwegian adolescent males: A focus group interview study

**DOI:** 10.3389/fpsyt.2022.1049336

**Published:** 2022-12-08

**Authors:** Sebastian Slotte, Hege Ramsøy-Halle, Line Melby, Jannike Kaasbøll

**Affiliations:** ^1^Department of Mental Health, Faculty of Medicine and Health Sciences, Regional Centre for Child and Youth Mental Health and Child Welfare (RKBU Central Norway), Norwegian University of Science and Technology (NTNU), Trondheim, Norway; ^2^SINTEF Digital, Department of Health Research, Trondheim, Norway

**Keywords:** help-seeking, facilitating factors, mental health, access to care, adolescent males, qualitative

## Abstract

**Background:**

Young males are overrepresented in suicide statistics and underrepresented in statistics of help-seeking and receiving help from formal health services compared with young females. Few studies have examined facilitating factors for help-seeking among adolescent males with no previous experience seeking mental health help. This study aimed to explore thoughts, attitudes, and experiences regarding facilitating factors toward formal help-seeking for mental health problems among Norwegian adolescent males.

**Method:**

Three focus group interviews were conducted, each including four adolescent males with no experience with help-seeking or receiving help from a help service for mental health problems. The interviews addressed topics such as barriers and facilitating factors for help-seeking and preferences regarding structural factors and modes of delivery of help. Data were analyzed according to the systematic text condensation method.

**Results:**

The analysis revealed three main categories of facilitating factors: (1) transparent information and available help services, (2) proactive and outreaching help services, and (3) the possibility for discreet help-seeking.

**Conclusions:**

Improving help-seeking is integral to accessing care and improving mental health. Help services can be more accessible and approachable for male adolescents if they offer discretion, the possibility to remain anonymous, the option to receive help in a convenient context, and outreach services.

## Background

Adolescent males have higher levels of mental health and psychosocial problems such as substance abuse and behavioral problems, i.e., oppositional-defiant disorder, conduct disorder, and attention-deficit and hyperactivity disorder (ADHD), and they are overrepresented in suicide statistics compared to adolescent females (1–3). Nevertheless, few adolescent males in need of treatment consult with or receive help from formal health care services, and adolescent males have more negative attitudes and intentions toward help seeking than females (4–8). A systematic review investigating whether online mental health services improve help-seeking for young people found that young males have been reported to seek less online help than females (9). Knowledge about barriers and specifically facilitating factors for seeking and receiving help for mental health and psychosocial problems among adolescent males is therefore needed (4, 5, 10). This study seeks to add knowledge to this gap.

Help-seeking behavior refers to actively seeking assistance from informal (e.g., family and friends) and formal (e.g., professionals) sources for acceptance, understanding, advice, information, treatment, and general support as a response to problems or distressing experiences (10). According to a model for youth help-seeking and service utilization developed by Srebnik et al. (11), there are three stages in the help-seeking pathway: (1) problem recognition, (2) decision to seek help, and (3) decision on the help source to turn to for help. These stages are elaborated in the conceptual framework of access to health care, both at the individual and system levels (12). Here, the initial help-seeking process concerns the health care need (approachability and ability to perceive), perception of needs and desire for care (ability to seek and acceptability), and health care seeking (ability to reach availability and accommodation).

According to systematic reviews on adolescent help-seeking, several studies have sought to understand why young people and adolescents do not seek professional help when they need it (13, 14). In general, adolescents may be reluctant to seek help due to their increased need for privacy and autonomy (15). For mild problems, adolescents in general typically turn to friends or the internet for assistance. For more serious problems, one systematic review and qualitative studies of adolescent help-seeking indicate that adolescents tend to turn to a trusted adult such as their parents or teachers (13, 16, 17). A recent systematic review on help-seeking behaviors for common mental health problems in adolescents concluded that stigma and negative beliefs about mental health services appear to be the most significant barriers to help-seeking for adolescents (13). The findings of an American representative survey (*n* = 6,510) on the perceived unmet needs for treatment among individuals with depression in the United States indicated that barriers to seeking help include concerns about cost, transportation or inconvenience, confidentiality, other people finding out, thinking they can handle the problem on their own and thinking that treatment will not help (18).

There is a well-documented gender difference in adolescents' help-seeking for mental health problems as well as perceived barriers to help-seeking (6). Studies on adolescent males' experiences with help-seeking indicate that they experience several complex barriers to help-seeking (17, 19–21). Qualitative interview studies found that young males expressed insecurity in talk therapy and challenges in recognizing and verbally expressing emotions, ADDIN EN.CITE (17, 19) and challenges when trying to navigate the health care system (17, 19). In addition, low mental health literacy (22, 23), conflicts with perceived gender-related expectations ADDIN EN.CITE (19, 21, 23–28) have been reported to be barriers for help-seeking in both quantitative and qualitative studies ADDIN EN.CITE (17, 19). Studies indicate that male adolescents are less likely to know where to access health services, and they have been reported to experience discomfort, embarrassment, fear, and shame when asking for help (20, 27, 28). A Norwegian web-based survey was conducted to investigate the effect of gender on help seeking for mental health problems in an adolescent population (*n* = 1,249) (6). The results showed that females were better able to identify psychological problems of anxiety and trauma, were more aware of mental health services, and perceived more barriers to seeking help (cost and waiting time) than males. According to the authors, gender differences in use of services might be attributed to different awareness of referral pathway services and parental influence on the help-seeking process.

Although the literature on facilitating factors for adolescent help-seeking for mental problems is scarce, the review of Aguirre et al. (13) identified previous positive experiences with health services and mental health literacy as facilitators. According to Radez et al. (29) review, positive attitudes and encouragement from young people's support networks and positive perceptions of contact with professionals were the most common facilitators. Moreover, a focus group study (30) on adolescents' views on help-seeking for emotional and behavioral problems found that a bond of trust with a help source was perceived as the main facilitator for the decision to seek help. Adolescent males and females shared more similarities than differences in the focus groups. Female participants mentioned formal service utilization (e.g., general practitioners and youth health care) more often than their male counterparts (30). In previous studies, male health care providers have been associated with increased use of adolescent programs by males (31). Although it is well known that early intervention is crucial to prevent the development of mental disorders, little is currently known about facilitators for help-seeking among adolescent males. Since adolescent males are underrepresented in mental health services and overrepresented insuicide statistics, it is crucial to identify the factors that encourage young adolescent males to seek help if needed. Hence, the overall aim of this study was to explore facilitating factors for seeking help for psychosocial problems from help services amongst adolescent males.

## Methods

### Study design, setting, and participants

The present study used a descriptive and explorative qualitative design utilizing focus-group interviews. Qualitative methods are valuable for identifying and characterizing the understanding and meaning of a particular phenomenon (32). Because focus-group interviews are dynamic, they provide insight into complex tasks and reveal factors influencing the topic (33). Focus groups may provide perspectives and information that are difficult to obtain from individual interviews (34). As a result of group discussions and the sharing of thoughts, participants may be able to come up with ideas and co-create thoughts they wouldn't be able to express in individual interviews (35, 36).

To develop the semi-structured interview guide, existing conceptual frameworks and empirical findings about adolescent help-seeking were reviewed. Furthermore, the interview guide and the moderator's role were also tested in a pilot-test focus group interview. The recruitment of pilot interview participants from schools was difficult, and only one male adolescent aged 17 years participated. It was evident from the participant and researcher's evaluations of the pilot interview that some questions were too similar, emphasizing the need for precision and probing questions.

Purposive sampling was used to recruit the informants. Eligible participants in this study were adolescent males (16–18 years) who had not received help for psychosocial problems from any formal help services. Principals at fifteen high schools (in a large Norwegian city) got contacted with information about the study. There were three high school principals who agreed to participate in the study. Recruitment of participants was delegated to the head of student services and environmental workers/social educators. A school employee registered adolescent males who were interested in participating in the study with their contact information. The adolescents were informed about the study and their contact information was returned to the first author once they agreed to participate. The researcher contacted potential participants on the list to verify their interest in participating in the study, and to determine if they met the inclusion criteria. Of these, six participated in the study. In addition, snowball sampling was used, where current research participants recruited six participants.

In total, 12 adolescent males between the ages of 16–18 years old (average age of 17 years) without experience with help-seeking or receiving help for psychosocial problems from help services participated. This study did not collect data about the participants' cultural or socioeconomic backgrounds. Despite that, commonalities between the participants were evident considering the inclusion criteria stating that the participants had to be between 16 and 18 years of age, be residents of Bergen municipality, attend high school, be able to speak Norwegian (passed level C2 course in Norwegian), and have no prior experience seeking or receiving from formal help services. These common features, therefore, suggest a relatively homogeneous sample in the study.

The focus groups were conducted at a conference room at Ung Arena Bergen in Bergen municipality in Norway. Ung Arena is a free municipal program for children and young people aged 12–25 offering low-threshold mental health and substance abuse services. Developed in collaboration with young people, the service is tailored to meet their needs. Ung Arena offers a variety of services, including drop-in conversations, follow-up, practical support, and social media contact. For children and young people, Ung Arena is described as “a place where you can come and talk about what's on your mind.” The data collection and the focus groups were held during the month of June 2020 in Bergen. In this period, Norway was affected by the Covid-19 pandemic, but the study was completed in line with the present infection control guidelines in Norway. Three focus group interviews were conducted. Each focus group interview consisted of four participants. The interviews were based on a semi-structured interview guide with some main topics to ensure room for flexibility in exploring emerging themes (Table 1). Participants were asked to read and sign informed consent forms before participating in focus groups.

**Table 1:** Main themes and questions in the interview guide.

**Main theme**	**Main question(s)**	**Potential follow-up themes/themes**
Problem definition: When does a problem become a problem?	*When do you define and acknowledge that challenges in your life become a problem you need to seek help for from a help service?*	Threshold for when to initiate help-seeking from a help service
		What attitudes and factors affect them
	*Have you ever experienced situations or problems where you have considered help-seeking from a help service?*	Examples and factors affecting them in this situation
		If no, what would you consider now if you felt the need to seek help from a help service?
		The threshold for when to initiate help-seeking from a help service
Barriers for help-seeking	*Which barriers do you experience toward help-seeking from a help service if you are in need of it? (experiences, thoughts, attitudes)*	Do you have examples of factors in your everyday life that make it harder for you to seek help from a help service? (context, environment, individual factors)
		Do you think that help-seeking from a help service would help if you don't feel good or you experience problems? If so, describe why.
		What do you think can help if you need help with psychosocial problems?
Facilitating factors for help-seeking	*What motivates you to seek help? (intention, motivation)*	Do you have examples from your everyday life that affect you positively toward help-seeking if you were to experience psychosocial problems?
		What facilitating factor is most important for you?
Barriers in relation to receiving help	*Which barriers do you experience in relation to receiving help from a help service if you were to experience psychosocial problems? Examples (factors, experiences, contextual factors, thoughts)*	Do you have examples or factors from your everyday life that make it more difficult for you to receive help from a help service for psychosocial problems?
		Attitudes about receiving help from help services for psychosocial problems
		What factor works as the biggest barrier toward receiving help for you?
		Are there bigger barriers to help-seeking or receiving help in itself?
Facilitating factors for receiving help from a help service	*What contributes to your motivation toward receiving help from a help service?*	Examples of everyday life that increase their likelihood of being able to or wanting to receive help.
		Factors regarding help services that are important for the motivation toward receiving help from them
Help services	*If you were to think freely, how do you want the help service to be if you were able to seek help from it if you experience psychosocial problems? (examples, factors)*	How can the help service be accessible when you consider or experience doubts about help-seeking?
		What is best when establishing the first contact?
		How should the help service be if you were to use it?
		Other important factors that can accommodate different barriers
		How can the help service be accessible when you consider or experience doubts about help-seeking?

The focus group interviews were moderated by the first author and were conducted in a meeting room at a low-threshold service, with each interview lasting between 1.5 and 2 h. The audio from the interviews was recorded and transcribed verbatim by the first author.

### Data analysis

The data were analyzed according to Malterud's systematic text condensation strategy (37). The method conveys a pragmatic and systematic approach to qualitative analysis, and the process consists of four steps. First, the transcripts from each interview were read by the authors to create an overall impression of each interview and to identify emerging topics and code groups. During the second phase of analysis, the first author read through the transcripts from each interview line by line to identify meaning units and relevant descriptive quotes and to create a coding list based on the identified themes. The analysis was conducted with flexibility, working back and forth between the different phases as meanings and topics changed. Each code group was divided into several subgroups through a discussion between the authors, and codes and quotes were consequently sorted among the subgroups. Condensates written in first person were developed for each focus group and compressed into one final condensate during the third phase of analysis. The condensate was synthesized and reconstructed by the first author during the fourth phase, including a discussion in the author group to increase the validation. The analysis was completed by rereading the transcripts to look for potential data that conflicted with the results.

## Results

Three themes described facilitating factors: (1) transparent information and available help services, (2) proactive and outreaching help services, and (3) the possibility for discreet help-seeking.

### Transparent information and available help services

Many of the participants highlighted a need to receive straightforward and transparent information about the help offered and how the help is given:

“*I think the threshold would be reduced by a sort of ... them giving more information about how it would be if seeking help. Like a “street-view” explanation, so you don*'*t have to go to the reception and be like: I have this and this problem, where do I go?”—Participant C1*

The informants emphasized that the help-seeking process should be as uncomplicated as possible and that open hours and location must convey a high degree of availability. Some adolescents expressed that they would prefer to be met by health care personnel (e.g., psychologists) who were relatively close to their age and who could relate to their current situation and context.

Online information was preferred by many participants/informants. One participant described how reading online that the service could help with specific problems such as his might reduce his threshold for help-seeking:

“*Back to what I said about that, you should be able to find all the information online. It would be smart if there were information about what sort of problems one can seek help for. Because that part about us not feeling that our problem is big enough, or that people can see; okay, I can see myself here, and there, and there, and then be like; “Maybe I do need help.””—Participant C1*

Participants explained that seeing positive experiences or results from friends and other people who have experienced similar problems and received help from a particular service was a facilitating factor for seeking help.

### Proactive and outreaching help services

The participants expressed a need for information about mental health problems, help-seeking, and relevant help services to be “pushed” on them, instead of having to look for it themselves. They envisioned that an outreach approach would be nice for catching up with those who might not be aware they needed help. One participant explained that information can challenge established attitudes and preconceptions about psychosocial problems and help services. Some of the participants expressed a lack of experience with being presented with this information in their natural context (e.g., school). They believe they would benefit from a visit from qualified staff presenting them with information, for example, during class at school:

“*And if you let another person in, to sort of use time, then it's like, this is important, and then you will be listening, and you get sort of “force-fed” the information. You don*'*t have to seek the information yourself. It is like, when you ask someone during math class about what they need help with, they will never be able to say that I need help with this and this because they don*'*t know what they are dealing with.”—Participant D3*

The participants highlighted that the most difficult part of the help-seeking process is to establish contact with the help service, particularly when they are initiating something that they perceived to be unknown or unsafe. One participant described how, for instance, an app for help-seeking that was for everyone could increase help-seeking, primarily because help-seeking would therefore be perceived as more normal and because you know where to start if you opt to seek help. They want to be offered opportunities to receive help from help services, and all pupils should receive such an offer. Some would have accepted an invitation like this, and some would consider it interesting to attend an appointment like this to talk about things, especially if they perceive it as normal:

“*I am sure this is about their* [various health care services] *capacity, but what if they just invite people in regularly, without you having to seek help yourself? That would have made it much easier. Because I think what people struggle with is to initiate contact with the help service, but if they can come to you, no matter what it's about, and ask if you would like to come, now it's your turn sort of ... Then the threshold would immediately be reduced. Because it changes from you experiencing a problem and having to initiate the contact to them checking up on you, and then it's sort of rude to say no. When you have made a deal, you feel obligated to follow up on it.”—Participant C3*

One participant emphasized that it would be easier to receive help if others offered him help, especially when he does not recognize his needs himself, and others do. Being offered help takes the responsibility to establish the first contact away from them, and according to them, this removes the barrier to defining a problem.

“*I agree, it seems like the help-seeking process is the biggest barrier, not receiving help. Because receiving help, you might not even have to ask about it. Someone might just help you when they detect that you are not feeling good. Whilst to reach out for help, you sort of must build enough courage to do it first, and then you might think of all of the things that can go wrong and stuff like that.”—Participant C1*

In general, several participants believed that the help would be more effective if it is received in contact with a helper in a physical location, compared to digital alternatives, especially if the help services are located physically close to their natural context. Some elaborated on how they would experience a lower threshold for help-seeking if the help service was located in many places around them, instead of being located at one central location, for instance, in school.

### Discreet help-seeking

The possibility of discreet help-seeking and being anonymous was expressed by the participants as a key facilitating factor that would lower the threshold for help-seeking. Anonymity would remove some of the barriers that the participants are experiencing by presenting themselves directly at a help services office, as exemplified by one participant:

“*You won't be held back by all of the things that you are afraid of when you get to “wear a mask.””—Participant C3*

Several participants thought it was easier to seek help online, especially if they could be anonymous. One participant argued that the fear of being perceived as abnormal or deviating would be less if communication took place through texts. Overall, the participants were positive about online help-seeking and anonymous chat channels. However, one participant thought the chat channels should be more directly linked to specialized help services, e.g., psychologists, as a part of the formal help services. It was also suggested that getting to know the helper from the help service online before meeting him or her in person could be a facilitating factor:

“*Generally, to have that possibility, because it is much easier for many to sit alone in their room, talking to someone, than to ask their mum: “Hey mum, can you drive me to the health care clinic because I want to talk to that person.””—Participant B3*

The informants expressed that it would be much easier to seek help if others did not need to see or know about it. They suggested that participating in different activities or approaching the help service for reasons other than help-seeking may increase discretion and facilitate counseling. One participant explained how he does not have to define his problem to be able to participate in activities arranged by a help service in their location, in addition to seeking help from them.

## Discussion

The purpose of this study was to explore the thoughts, attitudes, and experiences regarding facilitating factors for seeking and receiving help for mental health problems from help services among adolescent males in Norway. The analysis revealed three main categories of facilitating factors: (1) transparent information and available help services, (2) proactive and outreaching help services, and (3) the possibility for discreet help-seeking.

### Transparent information and availability of help services

A key facilitating factor in the present study concerns youths' need for transparent information about the help-seeking process and relevant help services. This concurs with previous studies (19, 38). Several studies indicate that a central barrier to help-seeking among adolescent males involves insecurity and a sense of lack of control related to the help-seeking process (17, 19, 38). Therefore, for adolescents, thorough information about how to access the services and their content may increase help-seeking and may increase their level of efficacious help-seeking at the individual level (39, 40). In the current study, “youth-friendly” information and help services were suggested to facilitate help-seeking. Moreover, as male health care providers have been associated with increased use of adolescent services, help services may benefit from the conscious use of male service providers and/or young user representatives/health care personnel in the initial contact establishment in the help-seeking process.

Structural factors such as open hours with a high degree of availability and available transparent information may facilitate help-seeking behavior (14, 19, 38). This was also indicated in the present study. Furthermore, empowering approaches and alternatives in contact establishment and modes of delivery (17, 19, 41, 42) that give adolescent males autonomy (39, 40), for example, being able to get to know the helper online through a chat, were suggested to facilitate perceived access to care and help-seeking. This might increase adolescent males' ability to reach for help and may convey a larger degree of availability and accommodation (12). However, research on modes of delivery of help indicates that one of four would rather not seek help, despite being able to receive help online (39). Therefore, even if low-threshold services with excellent accessibility are created, some young boys will not seek help because of barriers beyond access to services.

### Proactive and outreaching help services and systematic offers of help

Our results indicate that male adolescents prefer that help services reach out to them rather than the other way around. Systematic offers of help, such as bringing all young people to a public health or school nurse, were suggested by the informants in the current study to help more adolescent males identify any problems they may be facing. As suggested by previous research, school-based programs aiming to increase mental health literacy in adolescent males (17, 19, 38, 40) could provide a better foundation for male adolescents to understand and express their problems or concerns (10) and potentially make it easier to decide if, when and where they should seek help. Further research on the effects of outreaching help services and school-based programs that aim to increase mental health literacy and subsequent help-seeking if needed among adolescent males is therefore necessary.

### The possibility for discreet help-seeking

In line with previous studies (13), the possibility for discreet help-seeking, e.g., through online chats, was highlighted as a central facilitating factor for help-seeking among the study participants. Stigma has been identified as one of the most important barriers to help-seeking among adolescent males (14, 17, 38, 41). Studies have also identified concerns about confidentiality and help-seeking being very “public” as barriers to seeking help (13, 38). Having access to online directories may provide discreet pathways to face-to-face and online help for young people who need intensive services, as well as support the growing number of young people with mild or moderate mental health concerns (9). However, concerns have been raised about the quality of mental health information on the internet and the lack of involvement by mental health professionals (43). In the present study, the findings indicate the potential for better integration/connection between online services and the formal health care system. Future research should investigate the effectiveness of online mental health directories in facilitating young people's help-seeking needs and access to care, as well as integrated online and face-to-face formal help services, including a gender perspective.

The findings of the current study and previous studies (12, 39, 40) suggest that possibilities for discreet help-seeking in the adolescent males' natural context (e.g., services being located in schools) may facilitate help-seeking. Moreover, having reasons for and opportunities to establish contact with a help service other than the purpose of seeking help (e.g., participating in activities arranged by the service) can also aid in the initial help-seeking process. Some studies suggest that males are socialized to be goal-oriented and independent (44, 45) and that depending on others during help-seeking contradicts gender-related norms. To provide male adolescents with discreet access to support without compromising their sense of masculinity, innovative outreach services and help-seeking techniques that provide them with discreet access to support may be beneficial (46).

### Limitations and strengths of the study

A limitation of the study is that it did not take mental health status, sociodemographic and cultural variables into account during recruitment and data collection. The group was relatively homogenous, and having similar backgrounds may have made it easier for them to recognize and identify with each other during data collection and could also be considered a strength in this study (34). However, this group composition and sample have limitations, and it may, for instance, be difficult for the participants to present opinions or thoughts that deviate from the common perceptions presented in the group (47). Furthermore, the sample is likely to be biased in that those who would come forward are likely to have higher mental health literacy, beliefs around acceptability of using mental health services and higher levels of shame/stigma and therefore may express different facilitating factors than those who would not have approached. Further research should investigate how these factors may influence adolescent help-seeking.

There are several strengths of focus groups as a research method. They provide a synergy effect that may generate data that would not be discovered in individual interviews. The data collection was based on a semi-structured interview guide, which may decrease the degree of openness to the lived experience of the participants, providing them with a framework where the group prioritizes relevant meanings considering the interview guide. Nevertheless, the strength of a structured interview guide is that it can provide focused in-depth data on a specific object or phenomenon. However, the focused interview might relegate or reduce the presentation of the subject's interpretations concerning the phenomenon.

The decision to seek help may be influenced by contextual factors such as gender, rurality, and cultural background. This study focused only on male adolescents. It is possible that young female adolescents would have provided similar responses, as confirmed in a previous focus group study (30) on adolescents' views about seeking help for emotional and behavioral problems. Rurality is another contextual factor that may influence adolescent help-seeking. The present study involved participants who lived in or near a city. Several studies indicate that rural adolescents face additional barriers for obtaining professional psychological help because services are less accessible and available. Norway is considered one of the most gender-equal countries in the world, and the current study included Norwegian adolescents who were fluent in the Norwegian language. Ethnic minority male adolescents may have expressed other help-seeking factors. To disentangle such mechanisms, further research on facilitating factors for help-seeking should utilize representative population studies that use standardized measurements.

## Conclusion

The study presents facilitating factors for help-seeking among adolescent males who have not sought, or received, help for mental health problems from help services. The findings suggest that male adolescents may experience a greater amount of accessibility and approachability if the help services can offer discretion, the possibility to remain anonymous, the option to receive help in a context that is convenient for them, and outreaching help services. Utilizing the capabilities of technology to tailor services specifically for young men may increase the number of young men seeking both online and face-to-face support.

## Data availability statement

This article presents a dataset that is not readily available since participants have not consented to their data being shared publicly. Accordingly, the Regional Committees for Medical and Health Research Ethics have restricted the public sharing of the data.

## Ethics statement

The studies involving human participants were reviewed and approved by the regional committees for medical and health research ethics (REK) assessed the study and determined that the project did not fall within the committee's mandate related to the Health Research Act (ref: 2020/124675/REK sør-øst). The study protocol was then approved by the Norwegian Center for Research Data (ref: 486454). The patients/participants provided their written informed consent to participate in this study.

## Author contributions

SS performed the data acquisition. JK drafted the manuscript. Throughout the research process, all authors collaborated closely and contributed to data analysis and manuscript editing. The final manuscript was read and approved by all authors.
